# How do Regulatory T Cells Work?

**DOI:** 10.1111/j.1365-3083.2009.02308.x

**Published:** 2009-10

**Authors:** A Corthay

**Affiliations:** Centre for Immune Regulation, Institute of Immunology, University of Oslo and Oslo University HospitalRikshospitalet, Oslo, Norway

## Abstract

CD4^+^ T cells are commonly divided into regulatory T (Treg) cells and conventional T helper (Th) cells. Th cells control adaptive immunity against pathogens and cancer by activating other effector immune cells. Treg cells are defined as CD4^+^ T cells in charge of suppressing potentially deleterious activities of Th cells. This review briefly summarizes the current knowledge in the Treg field and defines some key questions that remain to be answered. Suggested functions for Treg cells include: prevention of autoimmune diseases by maintaining self-tolerance; suppression of allergy, asthma and pathogen-induced immunopathology; feto-maternal tolerance; and oral tolerance. Identification of Treg cells remains problematic, because accumulating evidence suggests that all the presently-used Treg markers (CD25, CTLA-4, GITR, LAG-3, CD127 and Foxp3) represent general T-cell activation markers, rather than being truly Treg-specific. Treg-cell activation is antigen-specific, which implies that suppressive activities of Treg cells are antigen-dependent. It has been proposed that Treg cells would be self-reactive, but extensive TCR repertoire analysis suggests that self-reactivity may be the exception rather than the rule. The classification of Treg cells as a separate lineage remains controversial because the ability to suppress is not an exclusive Treg property. Suppressive activities attributed to Treg cells may in reality, at least in some experimental settings, be exerted by conventional Th cell subsets, such as Th1, Th2, Th17 and T follicular (Tfh) cells. Recent reports have also demonstrated that Foxp3^+^ Treg cells may differentiate *in vivo* into conventional effector Th cells, with or without concomitant downregulation of Foxp3.

## Introduction

The concept of suppression mediated by T cells is nearly as old as the discovery of T cells as a separate lineage of lymphocytes. Already in the early 1970s, it was proposed that suppressor T cells would be capable of inhibiting other T cells, and thereby mediate immunological tolerance and self/non-self discrimination [1–3]. Suppressor T cells, which were characterized by expression of the CD8 (Lyt-2) cell surface marker, have been the topic of more than 1000 scientific publications. However, the existence of suppressor T cells as a distinct lineage of T cells has been very controversial [4]. In fact, the concept of suppressor T cells was largely abandoned by the end of the 1980s, essentially because of the poor characterization of the cells and the lack of specific markers [4, 5].

In the mid-1990s, a new subpopulation of suppressor T cells was proposed which expressed CD4 and which was named regulatory T (Treg) cells [5]. Accordingly, CD4^+^ T cells are now commonly divided into two distinct lineages: Treg cells and conventional T helper (Th) cells. Conventional Th cells control the adaptive immunity by activating, in an antigen-specific fashion, other effector cells such as CD8^+^ cytotoxic T cells, B cells and macrophages. Treg cells are defined as T cells in charge of suppressing potentially deleterious activities of Th cells. Treg cells represent nowadays a large field of research and a long list of Treg-associated suppressive mechanisms have been reported [6, 7]. However, many central aspects of Treg cell biology remain obscure and hotly debated [8–18]. The present review will focus on CD4^+^ Treg cells and will not discuss the older literature on the functionally-related suppressor T cells. My main objective is to briefly summarize the current knowledge in the Treg field and to define some key questions which remain to be answered. I also would like to encourage all readers interested in immunologic controversies to read and send contributions to the discussion forum of the *Scandinavian Journal of Immunology* [14–23]*.*

## Seven key questions about Treg cells that remain to be answered

### What are the functions of Treg cells?

**Figure 1:**
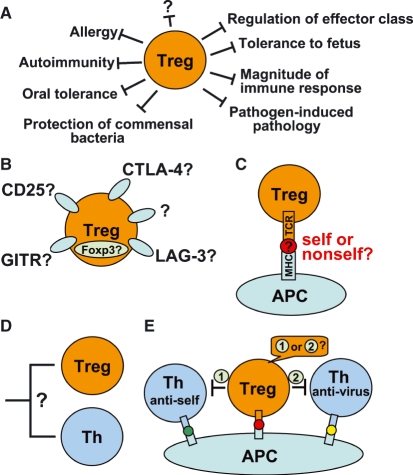
Five key questions about Treg cells. (A) What are the functions of Treg cells? (B) What molecular markers can be used to distinguish Treg cells from conventional Th cells? (C) Do Treg cells recognize self or non-self? (D) Do Treg cells represent a distinct lineage of CD4^+^ T cells? (E) How do Treg cells know which Th cell to suppress? In other words, how do Treg cells discriminate between the bad (i.e. self-reactive) Th cells, which should be suppressed, and the good (i.e. virus-specific) Th cells which should not?

The primary function of Treg cells was originally defined as prevention of autoimmune diseases by maintaining self-tolerance [24]. Over the years, several additional functions have been suggested and it will be important to clarify what Treg cells actually do in the immune system. Presently, at least 10 non-exclusive functions have been proposed for Treg cells (Fig. 1A):

1. Prevention of autoimmune diseases by establishing and maintaining immunologic self-tolerance [24–27].
2. Suppression of allergy and asthma [28–30].
3. Induction of tolerance against dietary antigens, i.e. oral tolerance [31–34].
4. Induction of maternal tolerance to the fetus [35].
5. Suppression of pathogen-induced immunopathology [36–38].
6. Regulation of the effector class of the immune response [10, 11].
7. Suppression of T-cell activation triggered by weak stimuli [39].
8. Feedback control of the magnitude of the immune response by effector Th cells [13, 40].
9. Protection of commensal bacteria from elimination by the immune system [14].
10. Prevention of T cells that have been stimulated by their true high-affinity agonist ligand from killing cells that only express low-affinity T-cell receptor (TCR) ligands such as the self peptide-major histocompatibility complex (MHC) molecule that positively selected the T cell [16].

It will be important to establish whether Treg cells are indeed performing all the above-listed functions. A related question is whether distinct subsets of Treg cells are responsible for the various suppressive activities.

### How to identify Treg cells?

Molecular markers are essential tools for defining and for analyzing a subpopulation of immune cells. The collapse of the suppressor T cells at the end of the 1980s was largely due to the failure to define specific markers for these cells [4].

The most widely used markers for Treg cells are (Fig. 1B):

- CD25 [24, 41].
- cytotoxic T lymphocyte-associated antigen 4 (CTLA-4) [42, 43].
- glucocorticoid-induced tumour necrosis factor receptor family-related gene (GITR) [44].
- lymphocyte activation gene-3 (LAG-3) [45].
- CD127 [46, 47].
- forkhead/winged-helix transcription factor box P3 (Foxp3) [48–50].

Unfortunately, accumulating evidence suggests that the above-listed markers are not strictly Treg-specific. Upon activation, all T cells express CD25, the α-chain of the interleukin-2 (IL-2) receptor [51–53], IL-2 being a T-cell growth factor which is important for T-cell clonal expansion. CTLA-4 is a negative regulator of T-cell activation, which is upregulated on all CD4^+^ and CD8^+^ T cells, 2–3 days following activation [52–54]. Similarly, the expression of GITR [52, 53, 55] and LAG-3 [56, 57] is induced in T cells upon activation. It has been suggested that CD127, the α chain of the IL-7 receptor, could be used to discriminate between CD127^low^ Treg cells and CD127^high^ conventional Th cells in humans [46, 47]. However, it has been recently reported that most CD4^+^ T cells downregulate CD127 upon activation [53, 58]. Furthermore, loss of CD127 is a characteristic feature of T follicular helper (Tfh) cells, which provide help for B cells, in human tonsils [59]. It has been reported that naïve, CD25-negative mouse CD4^+^ T cells do not upregulate Foxp3 when activated [48, 50, 60]. However, it is now well documented that most human CD4^+^ and CD8^+^ T cells transiently express Foxp3 upon activation [53, 61–65].

In conclusion, all the presently-used Treg markers (CD25, CTLA-4, GITR, CD127, LAG-3 and Foxp3) appear to be general T-cell activation markers. This observation strongly suggests that T-cell activation is required for T-cell mediated suppression. However, it also implies that the current Treg markers are not truly Treg-specific and therefore are not reliable for distinguishing Treg cells from activated conventional Th cells.

### Is suppression by Treg cells antigen-specific?

Treg cells, like all CD4^+^ T cells, possess a somatically-rearranged TCR, which allows specific recognition of antigenic peptides in the context of MHC class II molecules. Activation of conventional Th cells requires specific antigen recognition by the TCR and one would expect Treg cells to follow the same rule. To clarify this issue, it is helpful to consider separately (1) the antigen specificity of the Treg cell itself and (2) the antigen specificity of the conventional Th cell that is suppressed by the Treg cell.

Concerning the antigen specificity of the Treg cell itself, *in vitro* experiments have demonstrated that Treg cells need to be first activated via the TCR to become suppressive [66, 67], although this has been contested by others [68]. This implies that (1) Treg-cell activation is antigen-specific; and (2) the suppressive activity of Treg cells is triggered in an antigen-specific fashion. The same requirement for antigen seems to apply for Treg functions *in vivo*, since the proliferation of Treg cells in lymph nodes was shown to be antigen-dependent [52]. Furthermore, in the experimental autoimmune encephalomyelitis (EAE) mouse model for multiple sclerosis, myelin basic protein (MBP)-specific Treg cells were detected and protection was associated with specificity for MBP [69]. In the non-obese diabetic mouse model for type 1 diabetes, Treg cells specific for a pancreatic autoantigen were much more efficient at preventing diabetes than polyclonal Treg cells [70, 71]. It was further shown that pancreas-specific Treg cells could only prevent diabetes when the Treg antigen was present *in vivo* in the pancreas [72]. Finally, destructive autoimmune gastritis could be prevented by transfer of stomach-specific Treg cells, but not with polyclonal Treg cells [73].

Concerning the antigen specificity of the conventional Th cell that is suppressed by the Treg cell, the key question here is whether the Treg cell and the Th cell need to recognize the same antigen or not. *In vitro* mixed-cultures experiments have demonstrated that Treg cells activated by their cognate antigen can suppress the proliferation of conventional Th cells with different antigen specificities [66, 67]. *In vivo*, there is also evidence that Treg cells may suppress Th cells with other antigen specificities [71, 74, 75]. However, the issue is not settled yet, because there are also reports of antigen-specific suppression by Treg cell *in vivo* [75–77]. For instance, Treg cells specific for proteolipid protein (PLP) peptide PLP139-151 could prevent EAE induced by the same peptide or by another peptide (PLP178-191) from the same antigen [75]. By contrast, PLP-specific Treg cells were unable to prevent EAE induced by other immunogens such as myelin basic protein (MBP) or myelin oligodendrocyte glycoprotein (MOG). These results indicate that the suppressive functions by Treg cells may operate in an antigen-restricted manner *in vivo*.

In summary, suppression mediated by Treg cells is clearly antigen-dependent. The activation of Treg cells is antigen-specific, which implies that the suppressive activity of Treg cells is triggered in an antigen-specific fashion. Concerning the target cell, there is evidence that Treg cells may suppress Th cells with different antigen specificities. However, it is possible that suppression is more effective, and thereby physiologically more relevant, when the Treg cell and the suppressed Th cell have the same antigen specificity. To clarify these important issues, there is clearly a need for more *in vivo* studies with Treg cells with defined antigen specificities.

### Do Treg cells recognize self, non-self or both?

The ability to discriminate between self and non-self is a central property of conventional Th cells. During T-cell thymic development, TCRs are generated stochastically by somatic gene rearrangements. To prevent autoimmunity, T cells with self-reactive TCRs are purged from the repertoire by depletion [78] or functional inactivation, also called *anergy* [79]. Thus, the conventional Th repertoire is being selected for recognition of non-self [19]. What do Treg cells recognize: self, non-self or both (Fig. 1C)?

It has been proposed that Treg cells, which are in charge of maintaining self-tolerance, would be self-reactive [67]. Supporting this hypothesis, self-reactive Treg cells have been observed in various mouse models for autoimmune diseases. For instance, Treg cells specific for the insulin B chain [74] or for a pancreatic islet autoantigen [71, 72] protected against type 1 diabetes. Furthermore, the presence of MBP-specific [69] or PLP-specific [75] Treg cells was associated with prevention of EAE. However, the self-reactivity of the Treg repertoire has been challenged by reports of Treg cells recognizing foreign antigens from bacteria [38, 80], fungi [81], the protozoan parasite *Leishmania major* [82, 83], allergens [30] and alloantigens [84, 85].

It has been suggested that Treg cells would be generated in the thymus from precursor cells with a high affinity TCR for a self peptide. This hypothesis has received support from experiments with TCR-transgenic mice [86–88]. However, the interpretation of the data has been questioned by another report showing that the differentiation of Treg cells was not induced by a self-agonist ligand expressed in the thymus [89].

Several attempts have been made to measure the diversity and specificity of the TCR repertoire expressed by Treg cells. These studies, all based on gene-manipulated mice with a limited TCR repertoire, revealed a considerable (10–70%) overlap between the TCRs used by Treg cells and naïve Th cells [90–93]. Initial studies of the Treg repertoire, which focused on the most frequently found TCRs, concluded that CD25^+^ CD4^+^ Treg cells exhibit a high frequency of TCRs specific for self peptides [90, 91]. However, more recently, a much larger analysis of hundreds of TCRs, including infrequently used TCRs, found little evidence that the Treg population preferably recognized self antigens [93]. It was concluded that non-self antigens are the cognate specificities of Treg cells [93, 94]. Collectively, these data suggest that self-reactivity may be the exception rather than the rule in the Treg repertoire, as it is for conventional Th cells.

### Do Treg cells represent a distinct lineage of CD4^+^ T cells?

How strong is the case for classifying Treg cells as a separate lineage of CD4^+^ T cells distinct from conventional Th cells (Fig. 1D)? This issue may be considered either in terms of molecular markers or in functional terms. As discussed above, a truly Treg-specific molecular marker is still lacking. As a substitute, can biological activities be used to define the Treg lineage?

Functionally, Treg cells are characterized by being suppressor cells which only suppress and do not activate other Th cells. One could wonder whether Treg cells represent the only CD4^+^ T cells with suppressive functions. The answer to this question is clearly negative. The existence of four distinct subsets of conventional Th cells, which differ in terms of cytokine production and function, has now been firmly established: Th1 [95], Th2 [95], Th17 cells [96, 97] and T follicular helper (Tfh) cells [98, 99]. Conventional Th cells control the adaptive immunity by activating other effector cells such as CD8^+^ cytotoxic T cells, B cells and macrophages. However, effector Th cell subsets have also been shown to suppress each other. For instance, Th1 cells secrete interferon-γ (IFN-γ) that inhibits the proliferation of Th2 cells [100, 101]. IL-4, which is produced by Th2 cells, suppresses Th1 development and secretion of IFN-γ by Th1 cells [102, 103]. Both IFN-γ and IL-4 inhibit Th17 differentiation and the production of IL-17 by effector Th17 cells [96, 97]. IL-17, which is secreted by Th17 cells, suppresses Th1 differentiation and was recently shown to protect mice from Th1-driven colitis [104]. IL-21, which is produced by Th2, Th17 and Tfh cells, inhibits the differentiation of Th1 cells [105]. Th1, Th2 and Th17 cells may all produce IL-10, which suppresses proliferation and cytokine production by various T-cell subsets [12, 106–110]. Thus, the ability to suppress T cells is clearly not an exclusive property of Treg cells and all CD4^+^ T cells appear to exert various kinds of suppressive activities. Therefore, the key question is whether there is a special CD4^+^ T cell lineage, the Treg cells, which is dedicated to suppression, while conventional Th cells can both activate and suppress other T cells.

Transforming growth factor β (TGF-β) is produced by some Treg cells and has been suggested to be an important mediator of Treg-mediated suppression in the gut [111]. TGF-β may act as an immunosuppressive cytokine which for example inhibits the secretion of immunoglobulin (Ig) M, IgG1, IgG2a and IgG3 [112]. However, TGF-β can also be immunostimulatory. In particular, TGF-β has been shown to specifically induce IgA [112–114] and IgG2b [115] isotype switch in B cells. It has been proposed that TGFβ-producing CD4^+^ T cells may represent another Th subset (Th3) with both mucosal Th function and downregulatory properties for Th1 cells [31, 32]. Thus, the main role of TGF-β-secreting CD4^+^ T cells in mucosal regions may be to function as Tfh cells and to help B cells to produce IgA, rather than to exclusively immunosuppress. In humans, it has also been proposed that Treg cells producing both TGF-β and IL-10 may induce B cells to secrete IgG4 [116].

The concept that Treg cells would represent a distinct T-cell lineage with ‘suppressor only’ activities has been further challenged by recent studies demonstrating that Foxp3^+^ Treg cells in human peripheral blood and tonsils had the capacity to produce IL-17 upon activation [117–119]. IL-17 is a proinflammatory cytokine, which is typically produced by Th17 cells and which is believed to be important for immunity against extracellular bacteria [96, 97]. Like conventional Treg cells, IL-17–producing Treg cells strongly suppressed responder Th cell proliferation [117–119]. Foxp3^+^ Treg cells produced IL-17 when activated in the presence of the proinflammatory cytokines IL-1β and IL-6, whereas IL-17 secretion was inhibited by TGF-β [118]. IL-17^+^ Foxp3^+^ Treg clones were plastic enough to either secrete IL-17 or suppress, depending on the nature of the stimulus provided [118]. Collectively, these data suggest that the suppressive activities attributed to Treg cells may in reality, at least in some experimental settings, be exerted by conventional Th cell subsets such as Tfh and Th17 cells.

### What is the function of Foxp3?

The Foxp3 transcription factor is considered the most reliable marker for Treg cells [48–50, 60]. Tissue distribution analysis has shown that Foxp3 is mostly present in lymphoid tissues [120]. The expression of Foxp3 is highly restricted to αβ T cells, and almost undetectable in B cells, γδ T cells, natural killer (NK) cells, macrophages and dendritic cells (DC) [48, 49, 60, 120, 121]. The expression of Foxp3 is mostly restricted to CD4^+^ T cells, but some CD8^+^ T cells do express Foxp3 as well [60]. Contradictory data have been published on whether Foxp3 can be expressed by murine Th1 and Th2 cells [48, 120]. There is an imperfect overlap between the expression of Foxp3 and that of CD25, the classical marker for Treg cells. In mice, Foxp3 could be detected in both CD4^+^ CD25^+^ and CD4^+^ CD25^−^ T cells, but it was much more abundant in CD4^+^ CD25^+^ T cells [48, 49]. In the lymph nodes and spleen, most CD4^+^ CD25^+^ T cells expressed Foxp3, but there was also a population of Foxp3^+^ CD4^+^ T cells, which did not express CD25 [60, 121]. In the lungs, most Foxp3^+^ CD4^+^ T cells were negative for CD25 [60].

Foxp3 was originally suspected to be important for Treg functions because mutations in Foxp3 were found to be the cause of two severe multiorgan autoimmune syndromes in humans, namely XLAAD (X-linked autoimmunity-allergic dysregulation syndrome) and IPEX (immunodysregulation, polyendocrinopathy, enteropathy, X-linked syndrome) [122–124]. Similarly, mutant *scurfy* mice with a disrupted Foxp3 gene develop a fatal lymphoproliferative disorder and die within 4 weeks after birth [120]. T cell-specific ablation of Foxp3 resulted in a lymphoproliferative autoimmune syndrome identical to that observed in Foxp3-deficient mice [60]. Thus, Foxp3 is clearly essential for T-cell functions and defective Foxp3 leads to lethal immune dysregulation. However, association between a defective gene and severe immunopathology does not necessarily imply that the gene is specific for a distinct T-cell subset dedicated to immunosuppression. Mice lacking other key immunoregulatory molecules such as CTLA-4 [125, 126], TGF-β [127] and TGF-β receptor on T cells [128], all exhibit lethal lymphoproliferative phenotypes very similar to Foxp3-deficient mice.

Foxp3 was initially suggested to represent the ‘master regulator’ or ‘lineage-specification factor’ for the development of Treg cells [48, 49, 60], but this hypothesis has been challenged [129]. Experiments with mice expressing a fusion protein of non-functional Foxp3 and green fluorescent protein suggested that Foxp3 may be required for Treg functions but not for lineage commitment [130, 131]. Another study concluded that a higher level of regulation upstream of Foxp3 determines the Treg lineage [132]. Rather than being the ‘master regulator’ for the Treg lineage, it has been proposed that the function of Foxp3 would be to amplify and fix pre-established molecular features of Treg cells [130]. Continuous Foxp3 expression has been reported to be essential for maintenance of the developmentally established suppressive program in mature Treg cells in the periphery [133]. It has been suggested that expression of Foxp3 must be stabilized by epigenetic modification such as demethylation to allow the development of a permanent Treg cell lineage [134–136].

Although Foxp3 is a transcription factor, its exact function remains largely unknown. It has been suggested that Foxp3 may act as a repressor of transcription with the function of regulating the amplitude of the response of CD4^+^ T cells to activation [137]. It has also been proposed that all human CD4^+^ and CD8^+^ T cells may upregulate Foxp3 and acquire suppressive properties upon activation [65]. Genome-wide analysis has shown that Foxp3 binds to the promoter region of 700–1100 genes, many of those genes being associated with TCR signalling [138, 139]. A large number of Foxp3-bound genes were up- or down-regulated in Foxp3^+^ T cells, indicating that Foxp3 may act as both a transcriptional activator and repressor [138, 139].

The main evidence supporting Foxp3 as a critical factor for Treg functions comes from experiments showing that naïve T cells could be rendered suppressive by retroviral gene transfer of Foxp3 [48, 49]. However, in some experimental settings, Foxp3 did not seem to be absolutely required for suppressive activity. For instance, Treg cells generated *in vivo* by prolonged exposure to a harmless antigen did not express significant Foxp3 mRNA [140]. Similarly, Foxp3 was not expressed by T regulatory cells 1 (Tr1), a Treg subset which is induced by IL-10 and which produces IL-10 [141–143]. Furthermore, Foxp3 was not found in CD69^+^ CD25^−^ Treg cells isolated from tumour-bearing mice [144].

The idea of Treg-restricted expression of Foxp3 was challenged by experiments on the role of Treg cells during viral infections in mice. Treg cells have been suggested to suppress virus-specific immune responses to prevent immunopathology caused by excessive immune responses [36–38]. Surprisingly, depletion of Foxp3^+^ T cells resulted in impaired rather than increased immunity against herpes simplex virus and lymphocytic choriomeningitis virus [145]. These results suggest that Foxp3 may be expressed by a subset of effector T cells required for virus clearance [145]. In my opinion, these data on the antiviral function of Foxp3^+^ T cells are clearly in contradiction with the nature of Treg cells which are defined as suppressor cells only.

A series of recent reports has demonstrated that Foxp3^+^ Treg cells may differentiate into conventional effector Th cells, with or without concomitant downregulation of Foxp3. Treg cells induced by TGF-β*in vitro* were shown to lose Foxp3 expression and suppressive activity upon restimulation in the absence of TGF-β [134]. Transfer experiments of labelled Foxp3^+^ T cells into T-cell-deficient mice revealed that a large fraction (45–80 %) of the Treg cells had lost Foxp3 expression 4 weeks after transfer [146–148]. In the lymph nodes and spleen, some of the transferred Treg cells had differentiated into IFN-γ-producing Th1, IL-4-producing Th2 cells and IL-17-producing Th17 cells [133, 146, 148], and induced lung inflammatory disease in recipient mice [148]. In the Peyer’s patches, Foxp3^+^ Treg cells efficiently downregulated Foxp3 and differentiated into Tfh cells that provided help for IgA production by B cells [147]. In mice, IL-12 was shown to induce IFN-γ production by Foxp3^+^ Treg cells *in vitro*, even while Foxp3 expression remained [149]. Furthermore, IL-6 induced Foxp3 downregulation in Treg cells and reprogrammed Treg cells to become Th17 cells [150, 151]. As many as 25% of small intestinal Th17 cells had expressed Foxp3 at some stage of their development [152]. It has been proposed that Treg cells may differentiate into Th17 cells *in vivo* in the presence of inflammatory signals [151, 152]. Notably, the existence of T cells co-expressing Foxp3 and IL-17 has been reported both in mice and in humans [117–119, 152, 153]. Collectively, these data question the stability of the Treg cell lineage and suggest that Foxp3^+^ T cells may represent Th cells that are not fully differentiated. Furthermore, accumulating evidence indicates that Foxp3 may be expressed by Th cells that produce proinflammatory cytokines such as IFN-γ and IL-17.

### How do Treg cells know which Th cell to suppress?

A major challenge for the Treg field is to understand how Treg cells discriminate between the bad (i.e. self-reactive) Th cells, which should be suppressed, and the good (i.e. virus-specific) Th cells, which should not (Fig. 1E). If this distinction is not made, the host will be immunosuppressed and succumb to microbial infection or cancer. Several models have been proposed to solve this conundrum.

According to the *crossregulation model* proposed by Leon *et al.* [154, 155], suppression by Treg cells is antigen-specific. In this model that has received some experimental support [75–77], Treg cells are suggested to be autoreactive and to suppress conventional Th cells with the same antigen specificity. This allows Treg cells to mediate natural tolerance by ensuring self/non-self discrimination. The mechanism of suppression is proposed to be based on a three-partner interaction between the Treg cell, the Th cell to be suppressed, and the antigen-presenting cell (APC) [154, 155].

The *TCR signal strength model* by Baecher-Allan *et al.* [39] is based on the assumption that autoreactive T cells in the periphery have low-affinity TCRs because T cells expressing high-affinity TCRs for self antigens are deleted in the thymus. It is proposed that Treg cells suppress the physiologic activation of autoreactive T cells associated with low signal strength, while T cells activated during inflammatory responses associated with high signal strength are refractory to this mechanism of suppression [39]. This model was recently further developed by Beriou *et al.* who proposed that inflammation could drive Treg cells to lose suppressive activity and to secrete IL-17, thereby dampening suppression and promoting a pro-inflammatory milieu [118].

During microbial infections, conserved pathogen-associated molecular patterns bind to Toll-like receptors (TLRs) on immune cells. According to the *Toll-like receptor (TLR)-mediated blockade of Treg suppression model* by Pasare and Medzhitov [156], TLR-mediated activation of DC results in blockade of the suppressive activity of Treg cells, thereby allowing activation of pathogen-specific adaptive immune responses [156]. A similar model has been proposed by Sutmuller *et al.* [157], in which Treg cells are directly inactivated during infections, when microbial products bind to TLR2 on the surface of the Treg cells. A main problem with these two TLR-based models is that they imply that immune responses against pathogens should always be associated with autoimmunity, since microbial infections inactivate Treg cells that are in charge of maintaining peripheral T-cell tolerance.

Ingested antigens lead to the generation of Treg cells that secrete TGF-β, IL-4 and IL-10, rather than IFN-γ and are capable of influencing naïve T cells in their immediate environment to do the same [31]. According to the *effector class regulation model* by Matzinger [10, 11], DC can act as ‘temporal bridges’ to relay information from orally immunized Treg cells to naïve CD4^+^ T cells to regulate the effector class of the immune response. The orally immunized T cells use IL-4 and IL-10 to ‘educate’ DC, which in turn induce naïve T cells to produce the same cytokines as those produced by the orally immunized Treg cells. In this model, conversion of a naïve T cell occurs only if it can interact with the same DC, although not necessarily the same antigen, as the Treg cell. According to Matzinger, Treg cells do not represent a separate lineage of CD4^+^ T cells dedicated to suppression. Instead, Treg cells are proposed to correspond to new subsets of Th cells, which can both suppress and activate immune functions, such as IgA production by B cells [10, 11].

In an updated version of the *associative recognition of antigen (ARA) model*, Melvin Cohn [13] has recently proposed that Treg cells are not involved in self/non-self discrimination. Instead, the function of Treg cells (*T suppressor*) would be to feedback control the magnitude of immune responses by effector Th cells [13]. In this model, Treg cells are suggested to be specific for non-self and to suppress conventional Th cells with the same antigen specificity [13].

## Concluding remarks

A major outcome of the intensive research efforts on Treg cells has been to revive interest for suppression mediated by T cells, a neglected research area after the collapse of the suppressor T-cell hypothesis at the end of the 1980s. T-cell mediated suppression was so disregarded that conventional Th cells were often considered erroneously as ‘activators only’. However, the stimulatory activities of T cells need to be counterbalanced by suppressive mechanisms, in order to fine-tune immune responses and to prevent immunopathology. Intrinsic negative feedback loops are critically involved in the activation of all T cells and mice deficient for key immunoregulatory molecules such as CTLA-4 exhibit lethal lymphoproliferative disease [125, 126]. It is well established that conventional Th cell subsets suppress each other [96, 97, 100–105]. More recently, several studies have started to uncover the importance of suppression mediated by effector Th cells during immune responses against pathogens. For instance, the significance of secretion by Th1 cells of the immunosuppressive cytokine IL-10 is being recognized. IFN-γ is essential for control of many pathogens, but survival of the host often also depends on the secretion of IL-10. In mice infected by the protozoan parasite *Toxoplasma gondii*, it was found that essentially all of the IL-10 derived from conventional Th1 cells, the same cell population that displays effector function against the parasite [12]. Similarly, virus-specific Th1 cells were shown to exert simultaneously stimulatory (IFN-γ production) and inhibitory (IL-10 secretion) functions during acute influenza infection [110]. IL-10 produced by influenza-specific Th1 cells had a crucial role in suppressing excess inflammation and associated immunopathology [110].

In 1988, Göran Möller, who was one of the most respected and influential immunologists in Scandinavia, wrote for the *Scandinavian Journal of Immunology* an editorial entitled ‘Do Suppressor T Cells Exist?’. In this article, he summarized his point of view in one sentence: ‘I am not questioning the existence of suppressive phenomena or findings that T cells can mediate suppressive effects, but I am skeptical of the notion of suppressor T cells as a separate subpopulation of T cells’[4]. Möller’s main argument to reject the concept of suppressor T cells was the lack of specific markers [4]. Time has passed and suppressor T cells have been renamed Treg cells [5]. It is undisputable that much has been learned about the mechanisms of suppression mediated by T cells, as testified in this review. However, it is striking to realize that we are still lacking a truly specific molecular marker for Treg cells, despite considerable research efforts. Göran Möller died last year [158, 159] and one can therefore only speculate whether he would have written, 21 years later, a new editorial entitled: ‘Do Regulatory T Cells Exist?’.
